# Supplementary material for: High-Quality Resolution of the Outbreak-Related Zika Virus Genome and Discovery of New Viruses Using Ion Torrent-Based Metatranscriptomics
Source: Viruses. 2020 Jul 21;12(7):782. doi: 10.3390/v12070782 (PMC7411838; doi:10.3390/v12070782)
Supplement: Supplementary file 1 [file viruses-12-00782-s001.pdf]

SUPPLEMENTARY INFORMATION

High-quality resolution of outbreak-related Zika virus genome and discovery of new viruses using Ion Torrent-based metatranscriptomics

Silvia Ines Sardi[1], Rejane Hughes Carvalho[2], Luis Gustavo Carvalho Pacheco[1], João Paulo Pereira de Almeida[4], Emilia Maria Medeiros de Andrade Belitardo[3], Carina Silva Pinheiro[1], Gúbio Soares Campos[1] and Eric Roberto Guimaraes Rocha Aguiar[1,5*]

[1] Post-Graduate Program in Biotechnology, Instituto de Ciências da Saúde, Universidade Federal da Bahia, Salvador, Bahia, Brazil, sissardi@yahoo.com.br (S.I.S.); lgcpacheco@gmail.com (L.G.C.P.); carinasilvapinheiro@gmail.com (C.S.P.); gubiosoares@gmail.com (G.S.C.); ericgdp@gmail.com (E.R.G.R.A.);

[2] Instituto de Ciências da Saúde, Universidade Federal da Bahia, Salvador, Bahia, Brazil,: hughescv@gmail.com (R.H.C.);

[3] Post-Graduate Program in Immunology, Instituto de Ciências da Saúde, Universidade Federal da Bahia, Salvador, Bahia, Brazil,: emilia_mandrade@hotmail.com (E.M.M.A.B.);

[4] Department of Biochemistry and Immunology, Instituto de Ciências Biológicas, Universidade Federal de Minas Gerais, Belo Horizonte, Minas Gerais, Brazil, jpereiradealmeida.mg32@gmail.com (J.P.P.d.A.);

[5] Department of Biological Science (DCB), Center of Biotechnology and Genetics (CBG), State University of Santa Cruz (UESC), Rodovia Ilhéus-Itabuna km 16, Ilhéus, Bahia, 45652-900, Brazil; ericgdp@gmail.com

*Correspondence: ericgdp@gmail.com

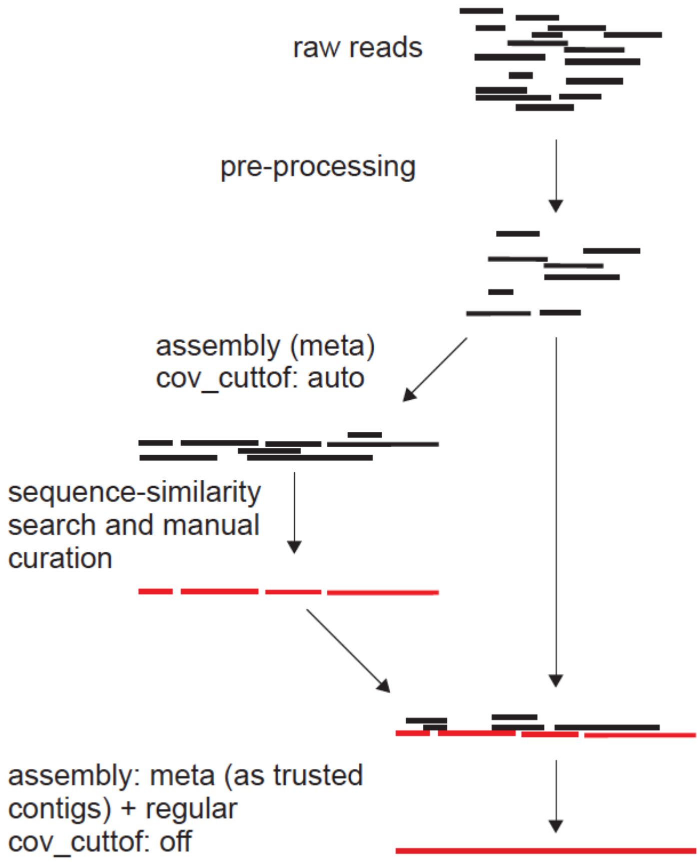

Supplementary Figure 1. Bioinformatics approach to reconstruct viral genomes based on ION torrent data. Raw reads are pre-processed in order to remove low quality reads, reads containing ambiguous bases and smaller than 70nt. Reads that passed in quality control were submitted to contaminants removal, which reads that matched host or bacterial genomes are filtered out. Pre-processed reads were used to *de novo* assembly contigs using "*meta*" strategy with automatic definition of coverage cuttof. Assembled contigs were characterized based on sequence-similarity searches and manually curated. Then, high quality viral contigs were used as anchors (trusted contigs) to perform posterior *de novo* assembly using regular "strategy" with coverage cuttof turned off.

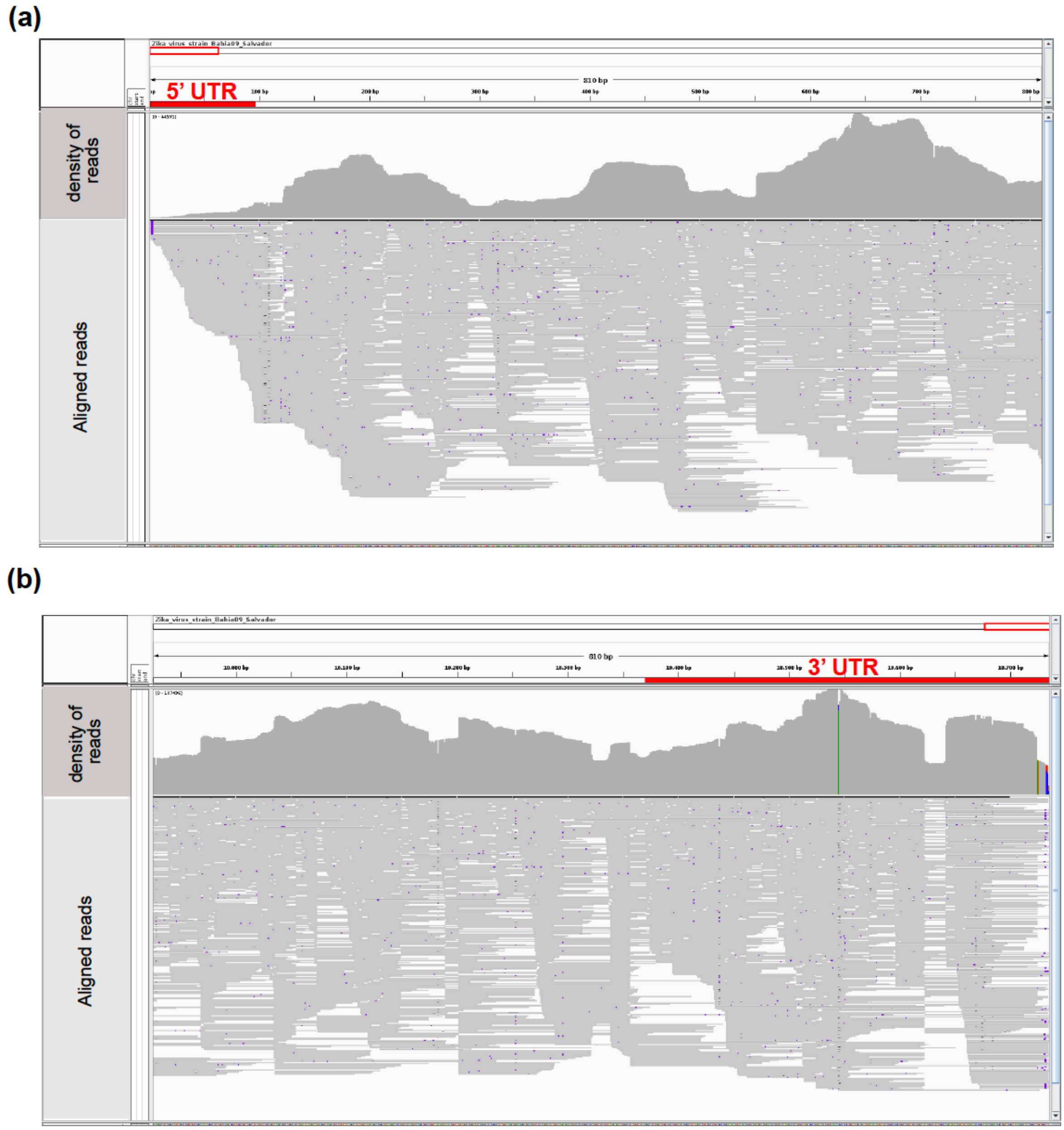

Supplementary Figure 2. Overview of reads aligned to UTR regions of the Zika virus genome assembled using IGV genome browser. Density and alignment profile of reads derived from 5′ (a) and 3′ (b) UTR regions of Zika virus genome. Red bars indicate UTR regions. A total of 6,784 reads were aligned to 5′ UTR and 250,906 reads to the 3′ UTR.

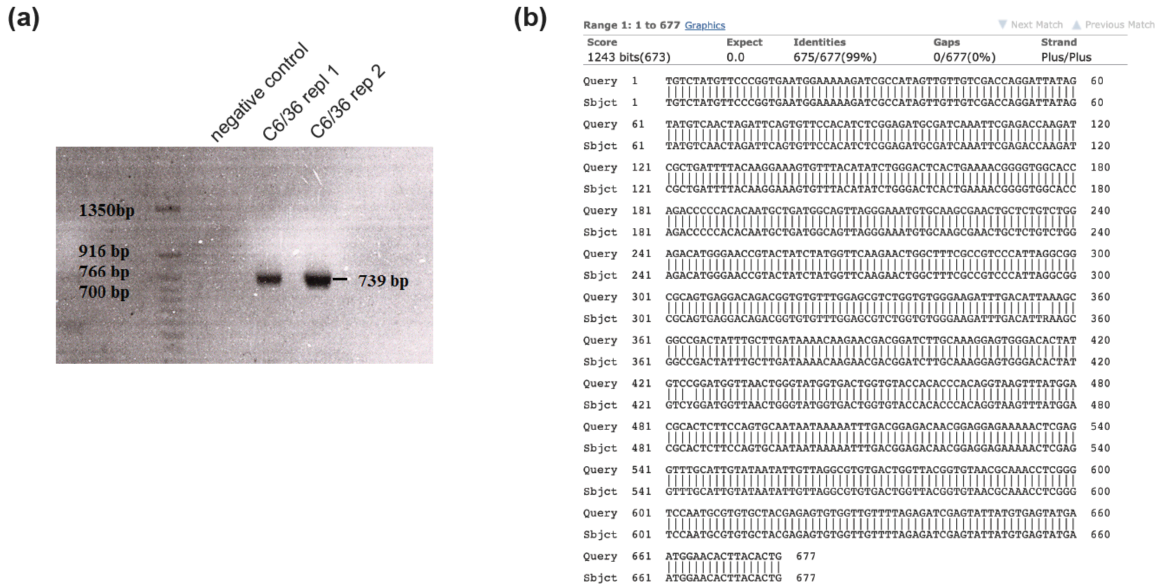

Supplementary Figure 3. Amplification of RdRp fragment of the new totivirus-like identified in *A. albopictus* C6/36 cell lines. A) RT-PCR of RdRp fragment of the new totivirus-like in uninfected C6/36 stock or water (negative control). B) Alignment of Sanger sequenced fragment and assembled contig derived from Ion torrent sequencing.
